# Competition or collaboration in regional Australia? A cross‐border and multi‐university approach to maximising rural health investments, community health and health workforce outcomes

**DOI:** 10.1111/ajr.12919

**Published:** 2022-09-12

**Authors:** Danielle White, Debra Jones, Pamela Harvey, Fiona Wright, Laura Tarrant, Louise Hodgetts, Kristy Allen, Steffanie Oxford, Andrina Mitcham, Kendall Livingstone

**Affiliations:** ^1^ Faculty of Medicine and Health, Broken Hill Rural Clinical School The University of Sydney Sydney NSW Australia; ^2^ Monash Rural Health Bendigo Vic. Australia; ^3^ Rural Health Mildura – Medicine Monash University Melbourne Vic. Australia

**Keywords:** collaboration, community based education, Governance, primary health care approaches, rural and remote education

## Abstract

**Aim:**

To describe the establishment of a cross‐border and multi‐university collaboration in rural Australia to mitigate potential competition, maximise Rural Health Multidisciplinary Training (RHMT) Programme investments and regional health workforce outcomes.

**Context:**

Rural Health Multidisciplinary Training programme investments have enabled the establishment of 19 Australian University Departments of Rural Health (UDRH) and 17 Rural Clinical Schools. The importance of these investments is acknowledged. However, in regional settings, due to limited clinical placement and training opportunities, there is potential for heightened competition between universities who are operating within shared geographical footprints. Competition between universities risks focusing RHMT programme activity on individual reporting requirements and activities, in preference to: regional needs; existing community–university relationships; and place‐based approaches to health workforce development.

**Participants:**

A rural New South Wales and Victorian RHMT‐funded departments, collectively known as the Sunraysia Collaboration.

**Approach:**

Strategic and operational processes, structures and actions underpinning collaboration formation and relationship consolidation will be described. Co‐design methodologies employed to collectively define collaboration vision and aims, governance framework and guiding principles, reporting structures and co‐contributions to teaching, research and service will be discussed. Collaboration sensitivity to the social, cultural, relationship and economic connectedness within the region and existing health workforce flows will also be explored.

**Conclusion:**

The Sunraysia collaboration demonstrates one approach towards mitigating potential competition between RHMT Programme funded universities within rural and remote Australia. The collaboration is an exemplar of co‐design in action providing an alternative approach to address RHMT Programme parameters and regional needs whilst supporting rural‐remote health workforce training and education innovations.

**What this paper adds:**

- Insight into the structures, processes and behaviours adopted to enable collaboration establishment and sustainability
- A discussion on the potential detrimental impacts of competition between RHMT programme funded universities in rural and remote locations
- Acknowledgement of the importance of understanding the potential implications of cross‐university competition on the relationship, social, cultural and economic contexts of rural and remote communities

**What is already known on this subject:**

- There are challenges in recruiting and retaining a rural and remote Australian health workforce
- Expanded RHMT programme investments have increased the potential of cross‐university competition within shared geographical footprints for clinical placements and detrimental impacts associated with competitive behaviours
- Cross‐organisational collaborations are identified as an effective approach to addressing complex challenges, particularly in rural and remote regions

## 1 INTRODUCTION

The complex challenges associated with the recruitment and retention of health professionals across rural and remote Australia are well documented.^1^ The Commonwealth Government has made significant investments through the Rural Health Multidisciplinary Training (RHMT) Programme in the establishment of nineteen University Departments of Rural Health (UDRH) and seventeen Rural Clinical Schools (RCS). These departments have carriage of multi‐disciplinary health student education and training within these regions.^2^, ^3^ Whilst the importance of these investments is acknowledged, limited clinical placement and training opportunities within these regions have the potential to heighten competition between RHMT‐funded universities operating within shared geographical footprints.^3^, ^4^ Competition risks focusing programme activity on individual university reporting requirements in preference to addressing regional needs, existing regional–university relationships, place‐based approaches to health workforce development and the efficient utilisation of programme resources.

The authors acknowledge the challenges associated with the establishment and sustainability of cross‐university collaborations given the competitive nature of these institutions. Universities can be characterised by competition for students, grants, academics and public and private resources.^4^ More recently, Australian universities have also experienced heightened competition in accessing the clinical placements required to address the education and registration needs of the increasing number of students enrolled in health degrees.^5^, ^6^ Whilst competition may be considered an inherent characteristic of contemporary universities, competition for scarce resources has been associated with cross‐university conflict and rivalry,^4^ strategic game‐playing, a decline in the sharing of information and innovations, the deformation of relationships and careless conduct.^7^ Limited evidence is available that describes the impacts of cross‐university competition on the social, educational, cultural, relationship and economic characteristics of regional, rural and remote Australian communities.

In contrast, the importance of strategic cross‐organisation collaborations as a means to resolve complex problems is increasingly acknowledged. These collaborations enable organisations to work collectively on defining challenges and implementing solutions that would be considered beyond the capacity of one organisation in isolation.^8^, ^9^, ^10^ These collaborations are perceived to have greater capacity to address regional challenges whilst also delivering mutually beneficial outcomes for partnering organisations and the communities in which collaborative efforts are delivered.^10^, ^11^, ^12^

This paper will describe the establishment of a cross‐border and multi‐university collaboration in rural Australia, the Sunraysia Collaboration. The collaboration seeks to: mitigate potential competition and the associated detrimental impacts of this behaviour between two RHMT‐funded universities located within a shared cross‐border region; maximise RHMT investments; and ensure programme parameters and regional community needs and expectations are met. Strategic and operational processes, structures and actions underpinning collaboration formation and relationship consolidation will be described. Co‐methodologies employed to collectively define the collaboration's vision and aims, governance framework and guiding principles, reporting structures and co‐contributions to teaching, research and service will be discussed. The collaboration's sensitivity to the social, cultural, relationship and economic connectedness within the region and the existing health workforce will also be explored.

## 2 DEFINING CROSS‐ORGANISATION COLLABORATIONS

Cross‐organisational collaborations are defined as ‘the linking or sharing of information, resources, activities, and capabilities by organizations in two or more sectors to achieve jointly an outcome that could not be achieved by organizations in one sector separately’.^10^ Features of successful cross‐organisation collaborations include: engagement that is focused on solving shared challenges and/or the realisation of common opportunities; the presence of core competencies across each organisation; alignment of the mission and aims of the collaboration to those of partnering organisations; acknowledgement that all partners bring valuable contributions to the collaboration; and value‐add potential for the collaboration, individual organisations and communities.^9^, ^10^, ^12^, ^13^

An extensive body of literature describes the outcomes and efficiencies associated with multi‐university research partnerships and university–industry partnerships. However, there is limited evidence that describes the frameworks or strategies that are required to establish and sustain multi‐university and multi‐disciplinary partnerships between University Departments or Rural Health within Australian rural and remote contexts that are focused on broader collaboration agendas.

Whilst the benefits of cross‐organisational collaborations have been described, a number of challenges to collaboration establishment and sustainability have also been identified. These challenges include: difficulties in achieving consensus on the goals of the collaboration; perceived loss of autonomy; coordination fatigue; time and effort investments required to establish trusting relationships; power imbalances; obstacles to performance and accountability; management complexity; and lack of collaboration sustainability when partners are confronted with changing environments.^10^, ^11^, ^13^ Despite these potential challenges, the authors propose that the Sunraysia Collaboration provides both RHMT‐funded universities with enhanced capacity to meet programme parameters and align health service and workforce strategies to the context in which the collaboration operates.

## 3 RHMT PROGRAMME GOAL AND PARAMETERS

The goal of the RHMT Programme is to improve the recruitment and retention of medical, nursing, dental and allied health professionals across rural and remote Australia, ultimately contributing to improved health and well‐being outcomes for populations residing in these regions.^2^ The programme is guided by seven parameters which include: the delivery of effective rural training experiences for health students from across disciplines; ensuring high‐quality rural training experiences; a focus on student selection and rural student recruitment; engagement with key partners and local communities to support student training; maintaining and progressing an evidence base and the rural health agenda; improvements to the health and wellbeing of Aboriginal and Torres Strait Islander people; and regional leadership in developing innovative training solutions to address rural workforce recruitment and retention challenges.^2^

## 4 COLLABORATION CONTEXT

The multi‐university collaboration brings together the Broken Hill UDRH, The University of Sydney and Monash Rural Nursing and Allied Health (RNAH), Monash University. Broken Hill UDRH is located in the south of Far West of New South Wales and Monash RNAH Mildura is located in North‐West, Victoria. Whilst these organisations are located in separate States, they share a similar geographical footprint. The interrelated nature of this footprint is reflected in family and social networks, cultural identity, organisational relationships, economic connectedness and existing health workforce flows that occur between the two States.^14^

A longstanding relationship had previously been established between strategic leaders from both organisations. This informal relationship provided a foundation to support the delivery of shared health workforce strategies that initially targeted regional secondary school student consideration and uptake of health career pathways. The consistency of strategic leadership from both organisations supported the creation of a shared sense of regional commitment, trusting and authentic relationships and features which are reported to be catalysts for the creation of successful cross‐organisational collaborations.^10^, ^12^, ^15^ The following co‐methodologies were then adopted to transition this informal relationship into a strategic multi‐university collaboration.

## 5 CO‐DEFINING THE NEED AND PERCEIVED BENEFIT OF COLLABORATION FORMATION

In late 2018, executive leaders from both organisations participated in a roundtable meeting to discuss collaboration establishment. This meeting was influenced by: an increased awareness across organisations of the shared health workforce and service complexities confronting the region; service access inequities associated with cross‐border health care provision; acknowledgement of the potential of a collaborative approach to amplify health workforce impacts; perceived value in leveraging the expertise, networks and relationships located within each organisation; receptiveness to social innovation and the pursuit of novel solutions; and enhanced capacity to deliver rewarding educational and sustainable placement opportunities by drawing on the academic, professional and infrastructure resources located across each organisation. Following this meeting a Collaboration Proposal paper was developed and provided to senior executives from both universities for consideration. Intent to Collaborate and Service Agreement documents were then developed with establishment of the collaboration officially endorsed by both universities in late 2019.

## 6 GOVERNANCE STRUCTURE AND GUIDING PRINCIPLES

An integrated Governance Framework was established to ensure the work undertaken by the collaboration did not inadvertently impact on the discrete work of each organisation. Executives from both organisations meet quarterly to provide strategic oversight of the activities undertaken on behalf of the collaboration. Guiding principles were also developed to inform the approach to be taken by the collaboration.^15^, ^16^ These principles included: a mutual recognition of the importance of collaborations in addressing the complex health and health workforce disadvantages and inequities experienced in the cross‐border region; developing a shared understanding of the purpose of the collaboration and realistic short, medium and long term goals; establishment and maintenance of trusting relationships between university partners, regional leaders and academics and between these entities and the communities in which they engage; and open and transparent monitoring, measuring, learning and sharing of the impacts and outcomes of the collaboration for university and community partners.

In addition to these principles, the following community engagement principles were also considered to be of importance given the context in which the collaboration was formed. These engagement principles focused on: active engagement with communities to identify unmet health and health workforce needs and solutions co‐design; respect for community leadership, autonomy, voice and choice; development of flexible strategies to align to local context, needs, relationships and resources; a focus on establishing and maintaining university–community trust and respect; the need for the collaboration to work with and alongside, communities/agencies in preference to doing things to or for them; and knowledge sharing to inform community and university decision‐making.^14^, ^17^

## 7 COLLABORATION ACTION AND IMPACTS TO DATE

Operational teams have been established to progress education and training innovations and research agendas of relevance to the collaboration and region. These teams are focused on: the development and delivery of student inter‐professional education that integrates high acuity and primary health care experiences; the further establishment of community–university partnerships to progress the co‐design of service‐learning innovations aligned to community needs; and the establishment of research projects that capture the work of the collaboration and workforce innovation impacts and outcomes.

To support this work, the collaboration has facilitated the re‐distribution of resources from both universities to enable: the appointment of co‐funded academic positions to progress service‐learning innovations; the establishment of student accommodation in more remote locations; provision of administration support to enable placement co‐ordination; support for student transport across the region; and the ongoing engagement of executive leaders.

The collaboration has: directly contributed to a growth in allied health and nursing student placements within the region; increased the number and diversity of host sites engaged in student placements; strengthened relationships with regional Aboriginal Health Services through shared networks and relationships; re‐focused approaches to the development of student placement opportunities through the adoption of co‐design methodologies; and extended the capacity of the region to engage in broader regional health workforce innovations such as the Far West Extended Nursing Placement Programme.^14^

## 8 REPORTING ON THE WORK OF THE COLLABORATION

The Governance Group acknowledged the importance of ensuring clear and transparent reporting to the Commonwealth and each university of the work undertaken by the collaboration. A reporting template was developed to capture collaboration activity aligned to RHMT Programme parameters. Both universities submit the same activity report as part of their RHMT requirements which clearly states that this is shared activity being reported by both universities through the collaboration.

## 9 DISCUSSION

The literature identifies that cross‐sector collaborations ‘are difficult to create and even more difficult to sustain because so much has to be in place or work well for them to succeed’ and that the ‘normal expectation ought to be that success will be very difficult to achieve’.^15^ Significant investments have been made by both The University of Sydney and Monash University to ensure the collaboration is underpinned by the evidence, structures, process and behaviours that are associated with successful cross‐organisational collaborations.^10^, ^11^, ^12^, ^15^, ^18^

The authors acknowledge the time commitment that has been required to co‐define the intent of the collaboration, co‐design the Governance Framework, co‐develop guiding principles, navigate the inter‐organisational processes required to transition the informal relationship to a formal and multi‐university endorsed collaboration and the importance of the re‐distribution of resources to enable Collaboration actions to occur.^18^

The COVID‐19 pandemic also created additional challenges for the collaboration in relation to the cross‐border approach adopted. This included border closures between NSW and Victoria, differing public health orders, concerns regarding the safety and well‐being of remote and Indigenous communities and well‐being of students entering these regions. Despite these implications, the commitment of the collaboration has been sustained and enhanced in alternative ways, ensuring sustainability and commitment to communities remain the focus.

Through the establishment of the collaboration and adoption of co‐design approaches, the collaboration has been able to maximise RHMT Programme investments within the region. This has included: the establishment of a well‐supported, co‐funded and locally embedded academic network; the development of service‐learning innovations that enhance the delivery of training to students and access to services of need for communities, including Aboriginal and Torres Strait Islander populations; the provision of structured rural placements that address university curriculum requirements and regional expectations; and student engagement in inter‐professional education experiences.^2^, ^3^, ^19^ The collaboration is now focusing on the development of a research strategy and evidence base to explore the efficacy of the collaboration, the rural training strategies being developed and delivered within the region and impacts for regional communities.^2^

In overcoming the inherent competitiveness of each institution, the collaboration seeks to refocus the intent of the work undertaken on the underpinning RHMT programme parameters and the health service and workforce needs and outcomes of regional, rural and remote communities located within the collaboration's footprint. The structures and processes described within this paper provide a framework and potential standards to inform the approaches adopted by other universities within shared geographical footprints that are seeking to establish regionally relevant and respectful relationships. As stated by Kennedy ‘the best hope is for a set of standards – cultural norms‐ that recognize that even in a highly competitive environment departures from fairness simply cannot be tolerated’.^20^

## 10 CONCLUSION

The Sunraysia collaboration demonstrates one approach towards mitigating potential competition between RHMT Programme funded universities within rural and remote Australia. The collaboration is an exemplar of co‐defining and co‐designing in action, provides an alternative approach to addressing RHMT Programme parameters and community needs whilst also supporting rural and remote health workforce training and education innovations. The collaboration recognises that solutions to rural health inequities and workforce challenges necessitate cross‐border, multi‐university and place‐based responses. The collaboration contributes to the effective utilisation of RHMT resources, academic and community expertise. Through the Sunraysia Collaboration, both universities can collectively meet RHMT Programme parameters whilst contributing to substantive and sustainable health service and workforce innovations at the regional level.

## AUTHOR CONTRIBUTIONS

DW: conceptualization; visualization; writing ‐ original draft; writing ‐ review and editing; final draft. DJ: conceptualization; visualization; writing – original draft; writing – review and editing. PH: conceptualization; visualization; writing – review and editing. FW: conceptualization; visualization; writing – review and editing. LT: conceptualization; writing – review and editing. LH: conceptualization; writing – review and editing. KA: conceptualization; writing – review and editing. SO: conceptualization; writing – review and editing. AM: conceptualization; writing – review and editing. KL: conceptualization; writing – review and editing.

## FUNDING INFORMATION

No external funding has been received to support the design and implementation of the Sunraysia Collaboration. This manuscript has not been published in part or full elsewhere.

## CONFLICT OF INTEREST

The authors declare no conflict of interest.
